# Diagnosis and treatment of solid pseudopapillary tumor of the pancreas: experience of one single institution from Turkey

**DOI:** 10.1186/1477-7819-11-308

**Published:** 2013-12-01

**Authors:** Ayşe Yagcı, Savas Yakan, Ali Coskun, Nazif Erkan, Mehmet Yıldırım, Evrim Yalcın, Hakan Postacı

**Affiliations:** 1 Department of Pathology, M.D, SB Izmir Bozyaka Education and Research Hospital, Izmir, Turkey; 2 Department of Surgery, M.D, SB Izmir Bozyaka Education and Research Hospital, Izmir, Turkey

**Keywords:** Solid pseudopapillary neoplasia, Diagnosis, Treatment

## Abstract

**Background:**

Solid pseudopapillary neoplasia (SPN) of the pancreas is an extremely rare epithelial tumor of low malignant potential. SPN accounts for less than 1% to 2% of exocrine pancreatic tumors. The aim of this study is to report our experience with SPN of the pancreas. It includes a summary of the current literature to provide a reference for the management of this rare clinical entity.

**Methods:**

A retrospective analysis was performed of all patients diagnosed and treated for SPN in our hospital over the past 15 years (1998 to 2013). A database of the characteristics of these patients was developed, including age, gender, tumor location and size, treatment, and histopathological and immunohistochemical features.

**Results:**

During this time period, 255 patients with pancreatic malignancy (which does not include ampulla vateri, distal choledocal and duodenal tumor) were admitted to our department, only 10 of whom were diagnosed as having SPN (2.5%). Nine patients were women (90%) and one patient was a man (10%). Their median age was 38.8 years (range 18 to 71). The most common symptoms were abdominal pain and dullness. Seven patients (70%) presented with abdominal pain or abdominal dullness and three patient (30%) were asymptomatic with the diagnosis made by an incidental finding on routine examination. Abdominal computed tomography and/or magnetic resonance imaging showed the typical features of solid pseudopapillary neoplasm in six (60%) of the patients. Four patients underwent distal pancreatectomy with splenectomy, one patient underwent a total mass excision, and one patient underwent total pancreatic resection. Two required extended distal pancreatectomy with splenectomy. Two underwent spleen-preserving distal pancreatectomy.

**Conclusions:**

SPN is a rare neoplasm that primarily affects young women. The prognosis is favorable even in the presence of distant metastasis. Although surgical resection is generally curative, a close follow-up is advised in order to diagnose a local recurrence or distant metastasis and choose the proper therapeutic option for the patient.

## Background

Solid pseudopapillary neoplasia (SPN) of the pancreas is an extremely rare epithelial tumor of low malignant potential. SPN accounts for less than 1% to 2% of exocrine pancreatic tumors [1]. Until it was defined by the World Health Organization (WHO) in 1996 as ‘solid pseudopapillary tumor’ of the pancreas, this tumor was described by using various names including ‘solid cystic tumor’, ‘papillary cystic tumor’, ‘papillary epithelial neoplasia’, ‘solid and papillary epithelial neoplasia’, ‘papillary epithelial tumor’ and ‘Frantz’s tumor’, ‘solid and papillary tumor’, ‘solid-cysticpapillary epithelial neoplasm’, ‘benign or malignant papillary tumor of the pancreas’ [2]. These tumors typically occur in young women during the second to fourth decade of life and are histologically characterized by cystic areas and solid pseudopapillary arranged cells. The origin of these tumors is still a matter of controversy.

In this study, we report our experience with SPN of the pancreas and include a summary of the current literature to provide a reference for the management of this rare clinical entity.

## Methods

A retrospective analysis was carried out of all patients diagnosed and treated for SPN in our hospital over the past 15 years (1998 to 2013). A database of the characteristics of these patients was developed, including age, gender, tumor location (data were derived from radiological investigations or surgical records) and size (data were derived from radiological investigations or surgical records and finally confirmed by pathology), treatment (data were derived from the medical records, including the types of surgery), and histopathological and immunohistochemical features. Pre-operative fine needle aspiration cytology FNAC) was performed in one patient. All the patients who underwent resection were followed up every six months. The investigations performed included routine blood studies, chest X-ray, CA-19-9 level and either an ultrasound or computed tomography (CT) scan of the abdomen.

This study was approved by the Local Institutional Review Board of Izmir Bozyaka Education and research hospital.

## Results

**Figure 1:**
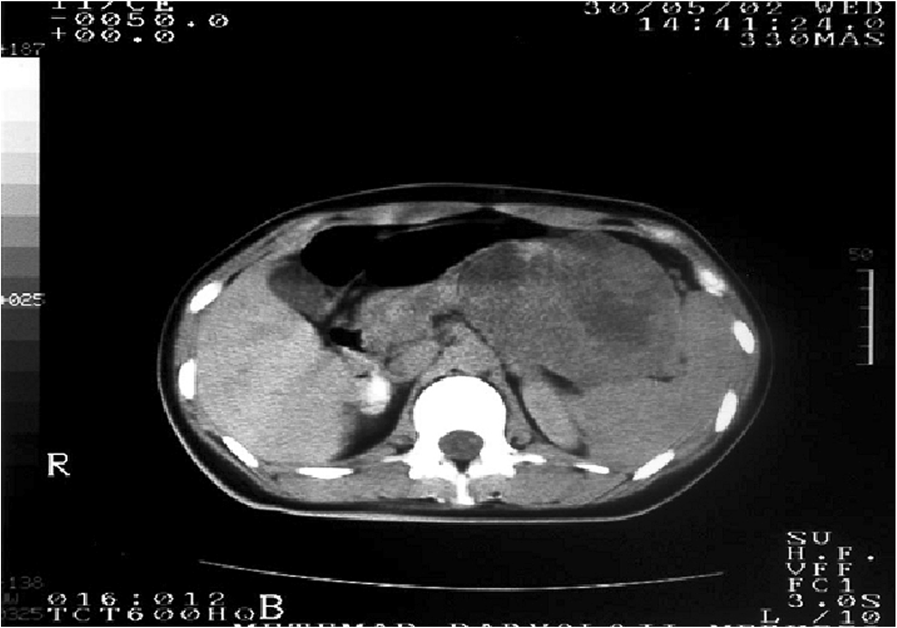
**Magnetic resonance imaging shows that the tumor is a well**-**marginated**, **large**, **encapsulated**, **solid and cystic mass with areas of hemorrhagic degeneration**, **as revealed by high signal intensity.**

During this time period, of 255 patients with pancreatic malignancy (which does not include ampulla vateri, distal choledocal and duodenal tumor) admitted to our department, only 10 were diagnosed as having SPN (2.5%). Nine patients were women (90%) and one patient was a man (10%). The patients had a median age of 38.8 years (range 18 to 71). The most common symptoms were abdominal pain and dullness. Seven patients (70%) presented with abdominal pain or abdominal dullness and three patient (30%) were asymptomatic with the diagnosis made by an incidental finding on routine examination. Abdominal CT and/or magnetic resonance imaging (MRI) showed the typical features of solid pseudopapillary neoplasm in six (60%) of the patients (Figure 1). Tumor markers (AFP, CEA, CA 19–9 and CA 125) were normal preoperatively in all patients. Usually, the tumors appeared as well-circumscribed lesions with a mixed cystic and solid component but were almost entirely solid or else cystic with thick walls. In one patient the tumor was located in the pancreatic head (10%), in four patients in the body (40%) and in the remaining five patients in the tail (50%). Four patients underwent distal pancreatectomy with splenectomy, one patient underwent a total mass excision and one patient underwent total pancreatic resection. Two required extended distal pancreatectomy with splenectomy. Two underwent spleen-preserving distal pancreatectomy. The mean diameter of the tumor was 8 cm (range 3 to 13 cm). Patient characteristics are summarized in Table 1.

**Table 1:** Patients characteristics

	**Patient 1**	**Patient 2**	**Patient 3**	**Patient 4**	**Patient 5**	**Patient 6**	**Patient 7**	**Patient 8**	**Patient 9**	**Patient 10**
**Age/Gender**	30/F	18/F	21/F	18/F	62/F	50/F	40/F	33/F	71/F	45/M
**Operation**	Distal pancreatectomy + splenectomy	Distal pancreatectomy + splenectomy	Total mass exicion	Spleen preser - ving distal pancrea -tectomy	Distal pancreatectomy + splenectomy	Subtotal distal pancreatectomy + splenectomy	Total pancrea- tectomy	Distal pancreatectomy + splenectomy	Subtotal distal pancreatectomy + splenectomy	Spleen preser - ving distal pancrea -tectomy
**Tumor location**	Body	Tail	Head	Tail	Tail	Body + Tail	Head + Body	Tail	Body + Tail	Tail
**Size(cm)**	9x7x5	13x9x6.5	13x6x5.5	6.5x5.7x3.6	4.5x3.5x3.2	12.5x11x6	4.3x3x3	4x3x2	11x7x7	3x3x2
**İnvasion**	(-)	Capsule and spleen	(-)	(-)	(-)	Capsule	(-)	(-)	Capsule	(-)
**Nodal status**	0/14	0/10	(-)	(-)	0/6	0/14	0/4	0/7	0/11	0/5
**Follow-up**	Healthy	Healthy	Healthy	Healthy	Healthy	7th month Liver and omental Metastasis 9th month exitus	29th day biliary and pancrea-tic fistula, 41th day exitus	Healthy	20th month Liver and omental Metastasis 24th month exitus	Healthy

**Figure 2:**
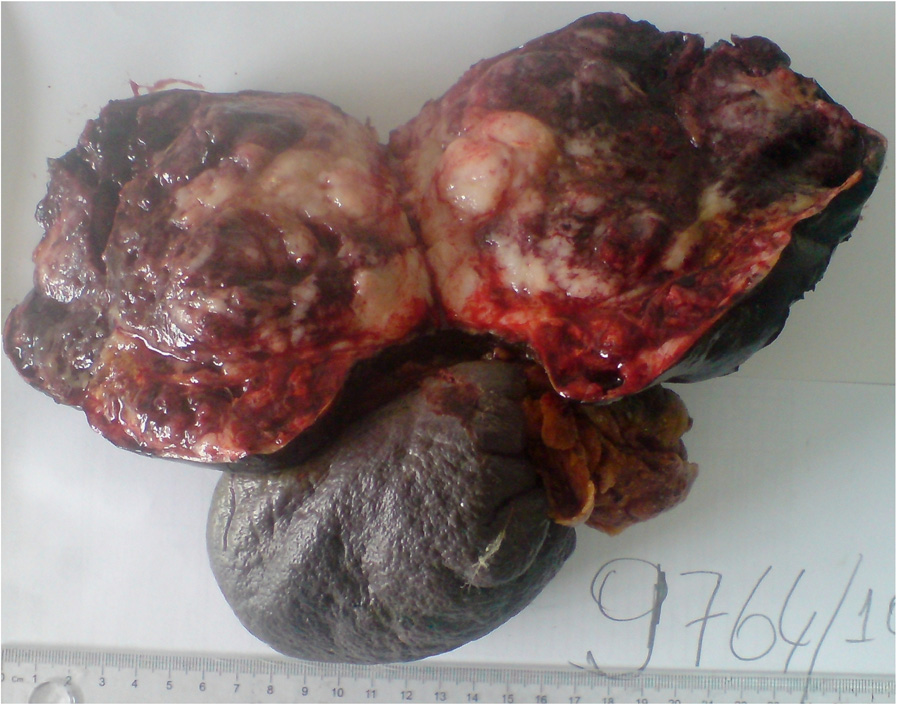
**Macroscopic appearance of distal pancreatectomy** **+ splenectomy specimen by SPN showing the solid and cystic component with hemorrhagic areas.** SPN, solid pseudopapillary neoplasia.

**Figure 3:**
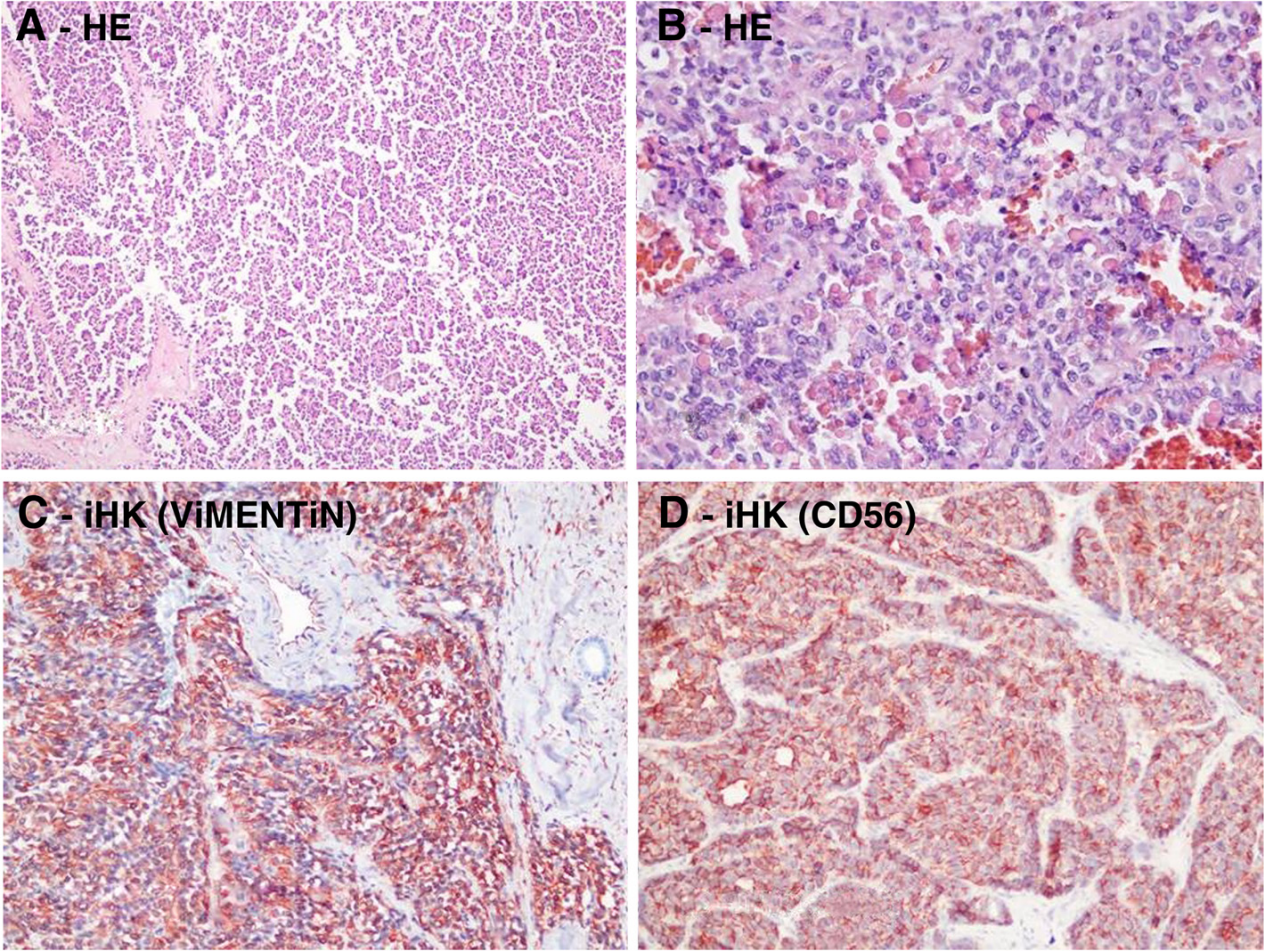
**Histologic appearance of solid pseudopapillar tumors. (A)** Solid pseudopapillar tumors exhibit a pseudopapillary pattern.** (B)** A portion of the tumor tissue shows a collection of hyaline globules. **(C)** Tumor cells typically show strong immunoreactivity for vimentin in the cytoplasm. **(D)** CD56 shows positive cytoplasmic membranous staining.

In eight cases lymph node dissection was done in a number between 4 and 14, whereas no dissection was needed for two patients. No lymph node metastasis was present in any patient. Macroscopically, there was diffuse hemorrhage and minimal necrosis between solid and cystic areas (Figure 2). At histopathological examination, tumor mass separated from pancreas with a fibrous capsula was seen. Pseudopapillary, cystic and solid growth patterns were seen in the tumor mass. Tumor cells had an ovally shaped, small and centrally localized nucleus and large eosinophilic cytoplasm. Tumors consisted of pseudopapillary structures made of cells aligned around fine vessels, solid areas, hemorrhagic areas and cystic areas of different size (Figure 3). No mitosis was seen in five cases, whereas minimal mitosis was present in two cases (2/10 per high powered field) and multiple mitosis were present in two cases (20/10 per high powered field; case numbers 6 and 9) (Table 2). The immunohistochemistry profiles are summarized in Table 3. Capsular invasion was present in three cases (case numbers 2, 6 and 9), spleen invasion was also present in case number 2. Along with capsular invasion, mitosis, nuclear polymorphism and necrosis were also significant in case numbers 6 and 9 at the time of diagnosis. These two cases were considered as malignant SPN and treated with six courses of gemcitabine + cis-platinum chemotherapy. Multiple liver and omentum metastases developed in case number 2 at the seventh postoperative month; this patient died at the ninth month. Multiple liver and omentum metastases developed in case number 9 at the 20th postoperative month and she died at the 24th month. The other eight cases have been followed up closely for an average of 7.9 years (between 1 and 16 years) and no recurrence or metastasis has been seen.

**Table 2:** Histopathologic features

	**Patient 1**	**Patient 2**	**Patient 3**	**Patient 4**	**Patient 5**	**Patient 6**	**Patient 7**	**Patient 8**	**Patient 9**	**Patient 10**
Development pattern	Solid, cystic, papillary	Cystic, papillary	Cystic, solid, papillary	Cystic, papillary	Cystic, solid, papillary	Solid, cystic, papillary	Solid, cystic, papillary	Solid, cystic, papillary	Solid, cystic, papillary	Cystic + papillary
Necrosis	(-)	minimal	minimal	(-)	(-)	(+)	(-)	(-)	(+)	(-)
Mitosis	(-)	(-)	2/10 per HPF	2/10 per HPF	2/10 per HPF	20/10 per HPF	(-)	(-)	20/10 per HPF	(-)
Pleomorphism	minimal	(-)	minimal	minimal	minimal	manifest	minimal	(-)	manifest	(-)

**Table 3:** The immunohistochemistry study

	**Patient 1**	**Patient 2**	**Patient 3**	**Patient 4**	**Patient 5**	**Patient 6**	**Patient 7**	**Patient 8**	**Patient 9**	**Patient 10**
Cytokeratin	Focal (+)	Focal (+)	Focal (+)	Focal (+)	Focal (+)	(-)	Focal (+)	(-)	(-)	(-)
CEA	(-)	(-)	(-)	(-)	(-)	(-)	(-)	(-)	(-)	(-)
Vimentin	(+)	(+)	(+)	(+)	(+)	Focal (+)	(+)	(+)	(+)	(+)
Chromogranin	(-)	(-)	(-)	(-)	Focal strong (+)	(-)	(-)	(-)	(-)	(-)
Neuron specific enolase	(+)	(+)	(+)	Focal slight (+)	(-)	(+)	(+)	(+)	(+)	(+)
CD10	(+)	Slight (+)	(+)	(+)	Focal (+)	(+)	(+)	(+)	(+)	(+)
CD56	Slight (+)	Slight (+)	(+)	(+)	(-)	(+)	(+)	(+)	(+)	(+)
Synaptophysin	Focal (+)	Focal (+)	Focal (+)	(-)	(+)	Slight focal (+)	Slight focal (+)	(-)	(-)	(-)
P53	Focal slight (+)	(-)	(-)	(-)	(-)	10% (+)	5% (+)	(-)	10% (+)	(-)
Kİ67	(-)	(-)	(-)	(-)	Under 1% (+)	10% (+)	3% (+)	(-)	10% (+)	(-)
Progesterone	(-)	(+)	(+)	(-)	(+)	(-)	(+)	(+)	(-)	(+)
EMA	Rare (+)	Rare cells (+)	Rare cells (+)	Rare cells (+)	(-)	Rare cells (+)	(-)	(-)	(-)	(-)

## Discussion

SPN is very rare; in fact, they only constitute about 5% of cystic pancreatic tumors and about 1 to 2% of exocrine pancreatic neoplasms [3]. They present mainly in the second and third decades of life [4]. Our series presented with a median age of 38.8 years, which is significantly older than in the literature (median age of 26 years) [5,6]. The origin of solid pseudopapillary tumors still remains unclear. These neoplasms have been suggested to have a ductal epithelial, neuroendocrine, multipotent primordial cell, or even an extra-pancreatic genital ridge angle-related cell origin [7].

The clinical presentation of the tumor is usually nonspecific. Abdominal discomfort or vague pain is the most common symptom, followed by a gradually enlarging mass and compression signs induced by the tumor. Some patients are completely asymptomatic, with the tumor detected incidentally by imaging studies or routine physical examination. Usually there is no evidence of pancreatic insufficiency, abnormal liver function tests, cholestasis, elevated pancreatic enzymes or an endocrine syndrome. Tumor markers are also generally unremarkable [4,8]. In our series, seven patients (70%) presented with abdominal pain or abdominal dullness, three patients (30%) were asymptomatic with the diagnosis made by an incidental finding on routine examination and preoperative tumor markers (AFP, CEA, CA 19–9 and CA 125) were within normal limits in all patients.

SPN can occur in every part of the pancreas but they are slightly more common in the tail [3]. Grossly, it appears as a large and encapsulated mass, generally well-demarcated from the remaining pancreas. In fact, invasion of the adjacent organs, such as the spleen or the duodenal wall, is rare. Depending on the tumor position (head, body or tail of the pancreas), the differential diagnosis includes adrenal mass, pancreatic endocrine tumor, liver cyst or tumor, or a pseudocyst [9].

Abdominal ultrasound and CT show a well encapsulated, complex mass with both solid and cystic components and displacement of nearby structures. There may be calcifications at the periphery of the mass and intravenous contrast enhancement inside the mass suggesting hemorrhagic necrosis [10]. However, when compared with MR imaging, CT has inherent limitations in showing certain tissue characteristics, such as hemorrhage, cystic degeneration, or the presence of a capsule. These features may, as shown at pathology, be suggestive of specific lesions such as SPN of the pancreas. Therefore, MR imaging may further aid in showing these characteristics and in the differential diagnosis of complex cystic masses within the pancreas [11]. Despite the technological improvements, preoperative diagnosis is difficult because of the similarity of findings among cystic lesions. Some studies advocate preoperative endosonography guided fine-needle aspiration biopsy for preoperative detection of the tumor, but this may not be accepted by others because of the uncertainty in diagnosis and the possible tumor spread [12,13]. In our series, preoperative endosonography guided fine-needle aspiration biopsy was performed in one out of ten patients and histology confirmed SPN.

In approximately 85% of the patients, SPN is limited to the pancreas, while about 10% to 15% of tumors have already metastasized at the time of presentation [14]. The most common sites for metastasis are the liver, regional lymph nodes, mesentery, omentum and peritoneum.

Once the diagnosis of SPN is made, surgery is the first choice of treatment. SPN is usually surrounded by a pseudocapsule and exhibits benign or low-grade malignancy. Conservative resection with preservation of as much pancreatic tissue as possible is the treatment of choice. According to the location of the tumor, distal pancreatectomy with or without splenectomy, pylorous preserving pancreatoduodenectomy, Whipple operation or enucleation can be performed. In our series, four patients underwent distal pancreatectomy with splenectomy, one patient underwent a total mass excision and one patient underwent total pancreatic resection. Two required extended distal pancreatectomy with splenectomy. Two underwent spleen-preserving distal pancreatectomy. Many studies have demonstrated that less aggressive surgical procedures could be preferred for the treatment of SPN [15]. Extensive lymphatic dissection or more radical approaches are not indicated when the disease is localized. Local invasion and metastases are not contraindications for resection. Portal vein resection is advocated when there is evidence of tumor invasion. For the metastases, surgical debulking should be performed, in contrast to other pancreatic malignancies. Metastases can be removed with enucleations or lobectomies and some patients with unresectable SPN may also have a long term survival [14]. The overall five-year survival rate of patients with SPN is about 95% [8].

Malignant SPN, designated as a solid-pseudopapillary carcinoma, occurs in 15% of adult patients. According to the WHO classification system, these are: 1) solid-pseudopapillary neoplasms with borderline malignancy potential; and 2) solid-pseudopapillary carcinomas. Criteria which distinguish potentially malignant tumors and which are classified as ‘SP carcinoma’ are: 1) angioinvasion; 2) perineural invasion; and 3) deep invasion of the surrounding pancreatic parenchyma. A recent study showed that some histological features, such as extensive necrosis, nuclear atypia, high mitotic rate, immunohistochemistry findings of expression of Ki-67 and sarcomatoid areas may be associated with aggressive behavior [16].

Adjuvant therapy is used only in a small number of patients because of the high resectability of SPN. The role of chemotherapy or chemoradiotherapy in the treatment of SPN is also unclear. In some studies, adjuvant chemotherapy and radiotherapy are reported in some unresectable cases with good results [17,18]. Neoadjuvant chemotherapy or chemoradiotherapy is also reported to have been successful in a few cases [19-22].

In the light of previous studies, our two patients (patients number 6 and 9) had capsular invasion besides significant mitosis (20/10 per HPF), nuclear pleomorphism and necrosis at the time of diagnosis and Ki-67 index was 10% (+). These two patients were accepted as having malignant SPN. They were given gemcitabine + cis-platinum chemotherapy. Multiple liver and omentum metastases developed in case number 2 at the seventh postoperative month; she died at the ninth postoperative month. Multiple liver and omentum metastases developed in case number 9 at the 20th postoperative month and she died at the 24th postoperative month.

## Conclusions

SPN is a rare neoplasm that primarily affects young women. The prognosis is favorable even in the presence of distant metastasis. Although surgical resection is generally curative, a close follow-up is advised in order to diagnose a local recurrence or distant metastasis and choose the proper therapeutic option for the patient.

## Abbreviations

SPN: Solid pseudopapillary neoplasia; HPF: High-power fields; CT: Abdominal computed tomography; MRI: Magnetic resonance imaging.

## Competing interests

The authors declare that they have no competing interests.

## Authors’ contributions

AY and SY participated in the data acquisition, data analysis, literature review and drafted the manuscript of this article. AC, NE and MY planned the analysis, participated in data acquisition, data analysis, literature review, patient treatment, and drafting and critical revision of the manuscript. EY and HP participated in immunohistochemisty and data analysis. All authors read and approved the final manuscript.
